# Ethylene Mediates Alkaline-Induced Rice Growth Inhibition by Negatively Regulating Plasma Membrane H^+^-ATPase Activity in Roots

**DOI:** 10.3389/fpls.2017.01839

**Published:** 2017-10-24

**Authors:** Haifei Chen, Quan Zhang, Hongmei Cai, Fangsen Xu

**Affiliations:** ^1^National Key Laboratory of Crop Genetic Improvement, Huazhong Agricultural University, Wuhan, China; ^2^Key Laboratory of Arable Land Conservation (Middle and Lower Reaches of Yangtze River), Ministry of Agriculture, Wuhan, China

**Keywords:** *Oryza sativa*, high-pH stress, growth inhibition, ethylene, H^+^-ATPase, ACC

## Abstract

pH is an important factor regulating plant growth. Here, we found that rice was better adapted to low pH than alkaline conditions, as its growth was severely inhibited at high pH, with shorter root length and an extreme biomass reduction. Under alkaline stress, the expression of genes for ethylene biosynthesis enzymes in rice roots was strongly induced by high pH and exogenous ethylene precursor ACC and ethylene overproduction in *etol1-1* mutant aggravated the alkaline stress-mediated inhibition of rice growth, especially for the root elongation with decreased cell length in root apical regions. Conversely, the ethylene perception antagonist silver (Ag^+^) and *ein2-1* mutants could partly alleviate the alkaline-induced root elongation inhibition. The H^+^-ATPase activity was extremely inhibited by alkaline stress and exogenous ACC. However, the H^+^-ATPase-mediated rhizosphere acidification was enhanced by exogenous Ag^+^, while H^+^ efflux on the root surface was extremely inhibited by exogenous ACC, suggesting that ethylene negatively regulated H^+^-ATPase activity under high-pH stress. Our results demonstrate that H^+^-ATPase is involved in ethylene-mediated inhibition of rice growth under alkaline stress.

## Introduction

Alkaline soils limit the survival of most plants and agricultural productivity. Worldwide, up to 434 million ha of land is alkalinized (Jin et al., 2006), becoming an environmental challenge for food production. High-pH stress inhibited plant growth by affecting numerous physiological processes including photosynthesis, ionic uptake and reactive oxygen species balance (Monshausen et al., 2007; Zhang and Mu, 2009).

Plant roots are the first organ that perceives soil alkalinity, which inhibits root elongation (Fuglsang et al., 2007; Xu et al., 2012; Li et al., 2015). H^+^-ATPase releases protons from cells to the apoplast, which has important roles in nutrient uptake (Palmgren, 2001; Krajinski et al., 2014), stomatal opening (Wang et al., 2014), polar transport of auxin and cell growth (Rober-Kleber et al., 2003). Acidification of the cell walls by H^+^-ATPase-mediated H^+^ excretion across the plasma membrane is critical for root elongation (Cosgrove, 2005; Sánchez-Rodríguez et al., 2010). A low apoplastic pH could increase the activity of expansins in the cell wall and cause cell expansion (Cosgrove, 2000). In the acid growth theory of plant cell elongation, auxin promotes cell elongation by regulating cell wall acidification, which may be attributed to the auxin-induced activation of H^+^-ATPases (Rayle and Cleland, 1992). High pH also drastically reduces the availability of many mineral nutrients in soil, thus imposing nutrient deficiency stress in plants. H^+^-ATPase-mediated proton extrusion is important for phosphate and iron uptake, as low rhizospheric pH can facilitate its solubilization (Palmgren, 2001; Zeng et al., 2012). Environmental stress also affects cell growth by changing H^+^-ATPase activity (Wu and Seliskar, 1998; Fuglsang et al., 2007). Several reports showed that H^+^-ATPase has a crucial role in the adaptation of plants to soil alkalinity. *Arabidopsis pks5* mutant plants were more tolerant to high pH, as PROTEIN KINASE SOS2-LIKE5 (PKS5) inhibits H^+^-ATPase activity by preventing interaction with the 14-3-3 protein (Fuglsang et al., 2007). In contrast, *j3* mutants were hypersensitive to alkaline stress, as Chaperone J3 activates plasma membrane H^+^-ATPase by repressing PKS5 (Yang et al., 2010).

Ethylene is involved in environmental stresses such as drought, salt, aluminum toxicity, phosphate, and boron deficiency (Sun et al., 2007; Lei et al., 2011; Martín-Rejano et al., 2011; Habben et al., 2014; Yang et al., 2015b). Aluminum toxicity and boron deficiency can induce rapid ethylene biosynthesis, which leads to inhibition of root elongation (Sun et al., 2007; Martín-Rejano et al., 2011; Tian et al., 2014). The biosynthesis of ethylene is regulated via successive enzymatic reactions: conversion of S-adenosyl-Met to ACC by 1-aminocyclopropane-1-carboxylic acid synthase (ACS) and conversion of ACC to ethylene by 1-aminocyclopropane-1-carboxylic acid oxidase (ACO) enzymes (Argueso et al., 2007; Lyzenga et al., 2012). In *Arabidopsis*, the ethylene signal is sensed by the membrane-bound receptors including ETR1, ETR2, ERS1, ERS2 and EIN4 (Sakai et al., 1998; Etheridge et al., 2006). Then, the signal from hormone perception in the endoplasmic reticulum to transcriptional regulation in the nucleus comprises CONSTITUTIVE TRIPLE RESPONSE1 (CTR1), ETHYLENE INSENSITIVE2 (EIN2), EIN3, and/or EIN3-LIKE1 (Guo and Ecker, 2004; Merchante et al., 2013). Several ethylene signaling genes have been identified and characterized in rice, including *OsERS1, OsETR2, OsCTRs, OsEIN2*, and *OsEIL1* (Ma et al., 2013; Yang et al., 2015a).

Root elongation in *Arabidopsis* is inhibited by exogenous ethylene or its precursor and apoplastic alkalization occurs in the elongation zone (Staal et al., 2011). Ethylene inhibits root growth primarily by stimulating auxin biosynthesis and modulating basipetal auxin transport toward the elongation zone (Rùzicka et al., 2007; Stepanova et al., 2007; Swarup et al., 2007). Ethylene up-regulates auxin biosynthesis in the root apex, leading to auxin accumulation and inhibition of root growth (Swarup et al., 2007). In addition, ethylene promotes basipetal auxin transport to affect root elongation by up-regulating *AUX1*, and the loss-of-function mutation of *AUX1* led to root ethylene insensitivity (Rùzicka et al., 2007; Li et al., 2015). PIN2 mediating auxin transport is required in the adaptation of plants to alkaline stress by modulating proton extrusion in the root tips to maintain root elongation (Xu et al., 2012). These observations suggest an important role of ethylene in regulating plant growth under alkaline stress.

For many lowland plants, extremely acidic soils with a pH less than 3 have been reported in their natural habitats, in which NH_4_^+^ is the dominant N form (Fyson, 2000). Rice (*Oryza sativa* L.) is the main staple food crop worldwide and is well adapted to NH_4_^+^ nutrition. Previous research suggested that the plasma membrane H^+^-ATPase is crucial for the adaptation of rice roots to low pH and may be partly responsible for the preference of rice to NH_4_^+^ nutrition (Zhu et al., 2009). And rice is more sensitive to alkaline stress than upland crops. Here, we report that strong growth inhibition under alkaline stress is regulated by ethylene-mediated H^+^-ATPase activity. Ethylene biosynthesis was up-regulated under alkaline conditions, inhibiting the activity of plasma membrane H^+^-ATPase and changing the apoplast acidification, resulting in the inhibition of root cell elongation under alkaline stress.

## Materials and Methods

### Plant Materials and Growth Conditions

Rice (Nipponbare, ZH11) and the published transgenic and mutant rice lines, including *eto1-1* (ZH11), *ein2-1* (Nipponbare) and OX-*A8* (Nipponbare), were used in this study. The *eto1-1* mutant was provided by Lizhong Xiong of the National Center of Plant Gene Research in Wuhan, China (Du et al., 2014). The OX-EIN2 and *ein2-1* line were provided by Jinsong Zhang of the Institute of Genetics and Developmental Biology of the Chinese Academy of Sciences in Beijing, China (Ma et al., 2013). The OX-*A8* line was provided by Yiyong Zhu of the Nanjing Agricultural University in Nanjing, China (Liu et al., 2012), and the expression of *OsA8* in OX-*A8* plants was significantly higher than WT (Supplementary Figure S1). The seeds were soaked in deionized water overnight at 30°C in darkness before transferring to a net floating on a 0.5 mM CaCl_2_ solution. After 7 days of germination, the plants were grown hydroponically in black pots containing a pH 5.5 nutrition solution of 1.44 mM NH_4_NO_3_, 0.3 mM NaH_2_PO_4_, 0.5 mM K_2_SO_4_, 1.0 mM CaCl_2_, 1.6 mM MgSO_4_, 0.17 mM Na_2_SiO_3_, 50 μM Fe-EDTA, 0.06 μM (NH_4_)_6_Mo_7_O_24_, 15 μM H_3_BO_3_, 8 μM MnCl_2_, 0.12 μM CuSO_4_, and 0.12 μM ZnSO_4_. After 7 days, the plants were transferred to a nutrition solution with a pH of 6, 7, or 8. Additionally, the rice plants were subjected to alkaline stress with or without the following additions: ethylene precursor ACC (1 μM, 10 μM), IAA (10 μM) and plasma membrane (PM) ATPase stimulator FC (10 μM). After 5 days in the various treatments, the phenotypes of plants were investigated. All experiments were performed with three replicates. The pH of the nutrition solution was measured daily using a pH electrode (METROHM) and regulated with 1 M HCl or KOH.

### Root Elongation Measurement and Medium Acidification Observation

To study the effect of alkaline stress on root growth and proton release, we grew rice seedlings on a one-half-strength Murashige and Skoog medium (MS). The seeds were surface sterilized for 15 min using 0.5% NaClO (w/v) and rinsed completely with ultrapure water. After incubation at 30°C for 3 days, the plants were planted on one-half-strength agarose solid MS medium at pH 6 or pH 8. The medium acidification under an alkaline condition (pH 8) with the ethylene precursor ACC (1 μM, 10 μM), AgNO_3_ (10 μM) and PM ATPase inhibitor VA (0.1 mM) was investigated by staining with the pH indicator bromocresol purple (0.04 g L^-1^).

### Measurement of H^+^-ATPase Activity and H^+^ Flux in Roots

After 3 days of treatment in different pH solutions, the PM H^+^-ATPase activity at the root tip was determined following the method of Zhu et al. (2009). The activity of H^+^-ATPase was measured by the Pi amount after 30 min of reaction in 0.5 ml reaction volume with 30 mM BTP/MES (pH 6.5), 5 mM MgSO_4_, 50 mM KCl, 1 mM NaN_3_, 5 mM ATP, and Brij 58 (0.02% w/v). The reaction was initiated by adding 5 μg of membrane protein for 30 min at 30°C and stopped using 1 ml of stopping solution containing H_2_SO_4_ (2% v/v), sodium dodecyl sulfate (5% w/v), and sodium molybdate (0.7% w/v), followed by 50 μl ascorbic acid (10% w/v). The color development of the phosphomolybdate complex was completed after 30 min. The absorbance at 820 nm was measured using a spectrophotometer.

The rice root H^+^ fluxes were measured at 10 mm from the root apex. The scanning ion-selective electrode technique was used to measure the root H^+^ fluxes with a Non-invasive Micro-test Technology system (NMT, Xuyue Beijing Sci. & Tech. Co., Ltd., Beijing, China). After the roots were equilibrated in the measurement solution for 20 min, they were immobilized in the measuring chamber in 5 ml of fresh measuring solution.

### Microscopic Analysis of Newly Grown Roots

After 5 days in various treatments, the new adventitious roots were imaged using a digital camera or an Olympus IX-70 microscope (Olympus, Japan). For anatomical analysis of the roots, toluidine blue staining of longitudinal cross sections (10 mm from apex) were performed to measure the cell sizes in the mature root zone. The size of the cortical cells was determined for 5 roots, with 10 cells each (*n* = 50).

### RNA Extraction, Reverse Transcription and Real-Time Quantitative PCR

The plants were grown hydroponically as described above for 3 days. The roots were harvested in three replicates, immediately frozen in liquid nitrogen and stored at -80°C. The total RNA was extracted independently using TRIZOL reagent (Invitrogen, Carlsbad, CA, United States). Reverse transcription was conducted using M-MLV Reverse Transcriptase (Promega, Madison, WI, United States). Real-time fluorescence quantitative PCR (RT-qPCR) for detecting the expression of the target genes was performed using a SYBR Green Real-Time PCR Master Mix Kit (TOYOBO, Japan) and CFX96TM Real-Time PCR Detection System (Bio-Rad, Hercules, CA, United States). The PCR conditions were as follows: 95°C for 5 min, 40 cycles of 95°C for 10 s, 60°C for 15 s and 72°C for 20 s. The relative mRNA levels for each gene in different samples were calculated as 2^-ΔΔC_t_^. Primer sequences used for the RT-qPCR are shown in Supplementary Table S1.

### Statistical Analysis

Each graphical plot represents the results of multiple independent duplicates from three replicates and the values are the means ± SE. Statistical significance between two treatments or two genotypes in a specific pH condition was used independent Student’s *t*-test. LSD test was used for multiple comparisons at the *p* < 0.05 level among three different treatments in a specific pH. Correlation coefficients were determined by Pearson’s bivariate correlation analysis at the *p* < 0.05 level.

## Results

### Response of Rice Growth to Alkaline Stress

**FIGURE 1:**
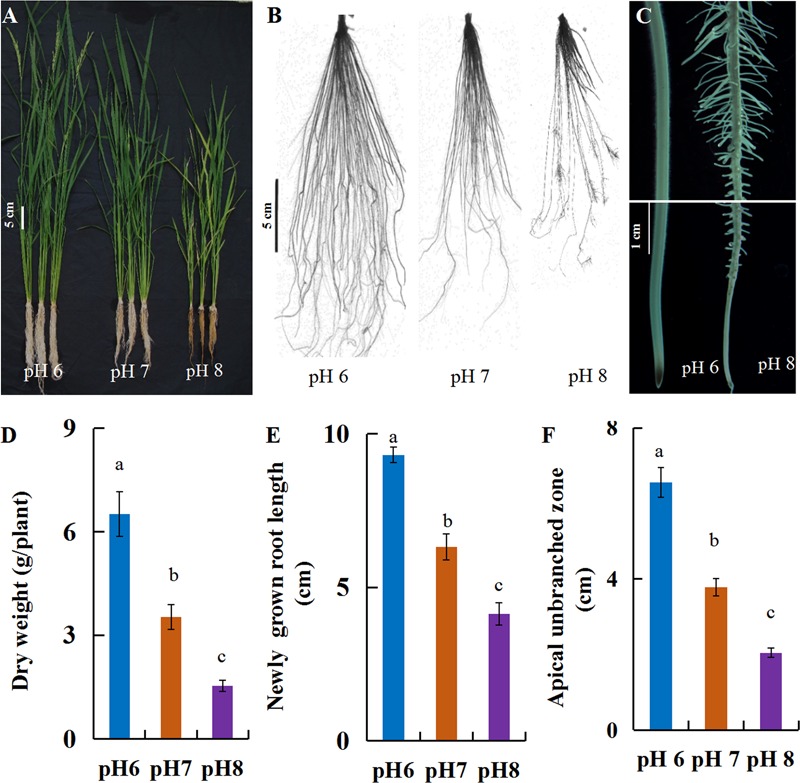
Phenotypic characterization of rice grown under alkaline conditions. Plants (cv. Nipponbare) were grown hydroponically for 7 days with full-strength nutrition at pH 5.5. The plants were transplanted into the full-strength nutrition at pH 6, 7, or 8. After 30 days, the plants were evaluated to investigate the phenotypic response to various pH. **(A)** Whole plants, **(B)** root morphology, **(C)** microscopy of newly grown roots after 7-day treatment, **(D)** dry weight per plant, **(E)** newly grown root length and **(F)** length of the apical unbranched zone. Values are the means (*n* = 6) and error bars denote the SE. Different letters represent statistical significance among treatments (*P* < 0.05).

In this study, a hydroponic system was used to investigate rice growth and root elongation under alkaline stress. After the seedlings were exposed to pH 6, 7 or 8 nutrition solutions for 30 days, the rice growth was severely inhibited by the alkaline stress (pH 8), with shorter plant height and root length than at pH 6 (**Figures 1A,B**), resulting in dramatic biomass reduction (**Figure 1D**). The plant root is the first organ that perceives soil alkalinity, which inhibits root elongation. The morphology of the newly grown roots was changed after short-term alkaline stress, with shortened root length, reduced apical unbranched zone and high density lateral roots in mature root zones (**Figures 1C,E,F**). These indicated that rice was more adaptive to an acidic condition.

The form of nitrogen (N) available to plants is an important factor that affects the plant rhizosphere pH by releasing H^+^ with ammonium uptake or OH^-^ with nitrate uptake. To verify the mediation of rhizosphere pH by the nitrogen form on rice growth, we investigated the phenotypes of rice growth on solid agarose solid medium supplemented with nitrate or ammonium at pH 6 or pH 8. After growing seedlings for 7 days, the medium with ammonium was acidified regardless of the medium pH treatments, and the medium with nitrate was alkalified by the roots (Supplementary Figure S2). After 15 days of growth on the medium, the rice plants grew well on the medium with ammonium, even at pH 8 (Supplementary Figure S2). These results suggest that the preference of rice to NH_4_^+^ nutrition was partly due to the adaptation of rice to an acidic rhizosphere environment.

### Ethylene Is Involved in Alkaline Stress-Mediated Inhibition of Plant Growth

**FIGURE 2:**
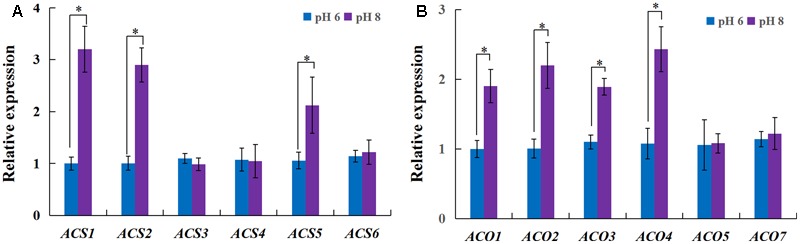
Expression of the genes encoding ethylene biosynthesis enzymes in adventitious roots. Plants (cv. Nipponbare) were grown hydroponically for 7 days in full-strength nutrition at pH 5.5. The plants were transplanted into full-strength nutrition at pH 6 or 8. After 3 days, the roots were evaluated to explore expression of the genes encoding ethylene biosynthesis enzymes. RT-qPCR analyses of the *ACS* genes **(A)** and the *ACO* genes **(B)**. Values are the means (*n* = 3), and error bars denote the SE. Asterisk indicates significance at the 10% level.

**FIGURE 3:**
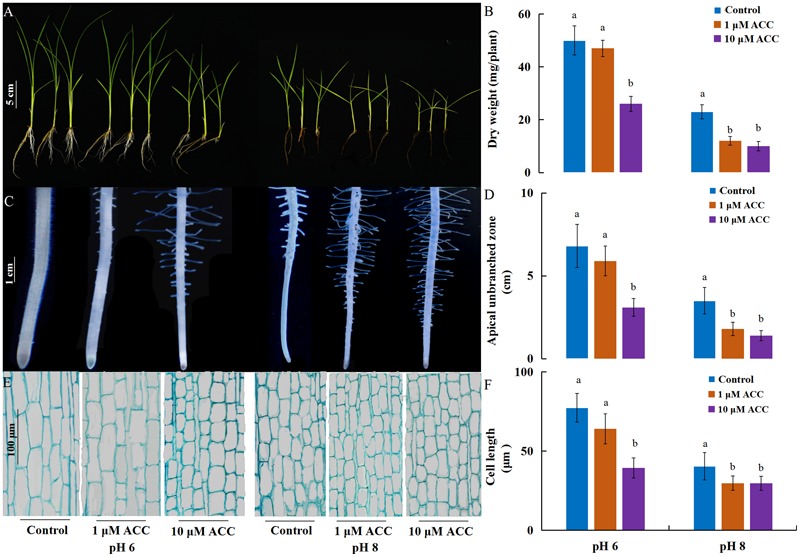
Effects of an ethylene precursor, ACC, on rice growth and root elongation under different pH conditions. After 5 days of germination, plants (cv. ZH11) were grown hydroponically under different pH conditions with 0, 1, and 10 μM ACC treatments. After 10 days, the plants phenotypes were investigated. **(A)** Whole plants grown under different treatments, **(B)** dry weight per plants (*n* = 6), **(C)** newly grown adventitious roots, **(D)** the length of the apical unbranched zone (*n* = 12), **(E)** paraffin section of the cell size in the root mature zone (10 mm from apex), and **(F)** average length of cortical cells (*n* = 50) in the root mature zone (10 mm from apex). Error bars denote the SE. Different letters represent statistical significance among treatments (*P* < 0.05).

As ethylene plays a role in the inhibition of primary root growth by affecting cell elongation (Swarup et al., 2007), we analyzed the effect of ethylene on root growth inhibition under alkaline stress. 1-aminocyclopropane-1-carboxylic acid synthase (ACS) and 1-aminocyclopropane-1-carboxylic acid oxidase (ACO) are two key enzymes responsible for ethylene biosynthesis in plants. The rice genome has six genes encoding ACS (*ACS1* to *ACS6*) and ACO (*ACO1* to *ACO5*, and ACO7 (Iwai et al., 2006). Among the *ACS* and *ACO* genes, *OsACS1, OsACS2, OsACS5, OsACS6* and *OsACO3, OsACO4, OsACO5* showed higher expression in roots than other genes and their expression levels were strongly induced by high pH, except *OsACS6* and *OsACO5* (**Figure 2**). We investigated the effect of 1-aminocyclopropane-1-carboxylic acid (ACC) on rice growth at normal pH (6) or high pH (8). The growth of plants was inhibited by exogenous ACC and the inhibition was aggravated under the alkaline condition (**Figure 3**). Under the normal pH condition, the growth of plants was not affected by 1 μM ACC compared with the control and the growth inhibition occurred up to an ACC concentration of 10 μM (**Figures 3A,B**). The plant growth was greatly suppressed by 1 μM ACC at the high pH condition (**Figures 3A,B**). The decreased plant dry weight could be attributed to the severe root development inhibition (Supplementary Figure S3). The length of the root apical unbranched zone was decreased by 54% with 10 μM ACC at pH 6 and up to 73% at pH 8 compared with the roots at pH 6 (**Figures 3C,D**). The cell length in the unbranched zone was reduced in the high pH condition and was aggravated by exogenous ACC (**Figures 3E,F**). These results suggest that up-regulation of *ACS* and *ACO* genes promoted ethylene biosynthesis and induced the suppression of plant growth under the high pH condition.

**FIGURE 4:**
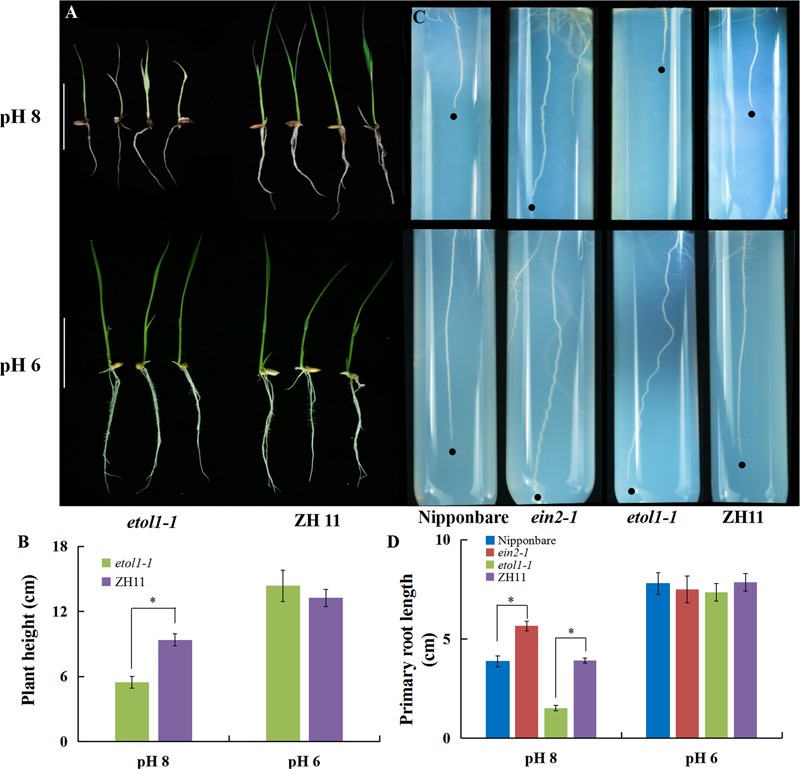
Ethylene is involved in alkaline stress-induced inhibition of rice growth. **(A)** Rice growth and **(B)** plant height of WT (cv. ZH11) and mutant *etol1-1* plants under different pH conditions in a hydroponic system. The ZH11 and *etol1-1* plants were grown hydroponically for 7 days under different pH conditions. To study the effect of alkaline stress on root elongation, the seeds were surface-sterilized for 15 min with 0.5% NaClO (w/v), rinsed completely with ultrapure water, incubated at 30°C for 3 days, and planted on solid agarose solid medium containing one-half-strength MS at pH 6 or 8. **(C)** The root elongation and **(D)** primary root length of WT plants (cv. Nipponbare and ZH11) and mutants (*ein2-1* and *eto1-1*) under alkaline stress. Values are the means (*n* = 6) and error bars denote the SE. Asterisk indicates significance at the 10% level.

To verify the effect of ethylene biosynthesis and signaling on alkaline stress-induced growth inhibition, a genetic approach was adopted using ethylene overproducer (*eto1-1*) mutants, and ethylene-insensitive mutants (*ein2-1*). In the *eto1-1* mutant, the stability of ACS2 is increased, which leads to overproduction of ethylene (Du et al., 2014). In the present study, we found that the *eto1-1* mutants showed similar growth to that of wild-type plants (ZH11) at normal pH (6). The growth of the *eto1-1* mutants was severely inhibited under an alkaline condition and displayed shorter roots and more serious etiolation in the shoot than the WT plants at high pH (**Figures 4A,B**). We also grew rice seedlings on one-half-strength Murashige and Skoog medium (MS) at pH 6 as a control and pH 8 for alkaline stress. Under high pH (8) stress, the *ein2-1* mutants had much longer roots than the WT plants (Nipponbare) and the roots were less inhibited than the roots at normal pH, but the root lengths of the *eto1-1* mutants were dramatically reduced compared with that of WT plants (**Figures 4C,D**). These results indicate that increased ethylene biosynthesis could be responsible for the inhibition of rice growth under high pH stress.

### Positive Effect of H^+^-ATPase on Rice Growth; Activity Influenced by External pH

**FIGURE 5:**
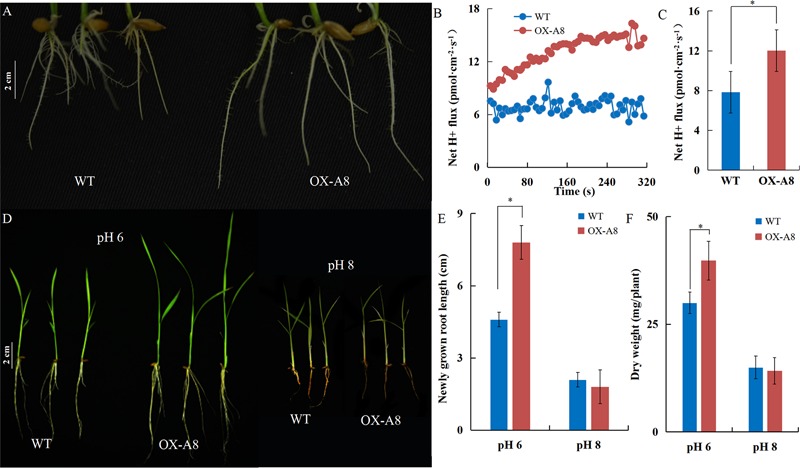
Positive effect of H^+^-ATPase on rice growth. After 7 days of germination, plants of Nipponbare (WT) and *OX-A8* plants were grown hydroponically for 7 days under different pH conditions. **(A)** Root growth of WT and *OX-A8* plants at pH 6. **(B,C)** Net H^+^ fluxes rate of WT and *OX-A8* plants on the root surface 10 mm from the root tips at pH 6. **(D)** The growth of WT and *OX-A8* plants at pH 6 or 8. **(E)** Newly grown root length and **(F)** dry weight per WT and *OX-A8* plant under different pH conditions. Values are the means and error bars denote the SE. Asterisk indicates significance at the 10% level.

**FIGURE 6:**
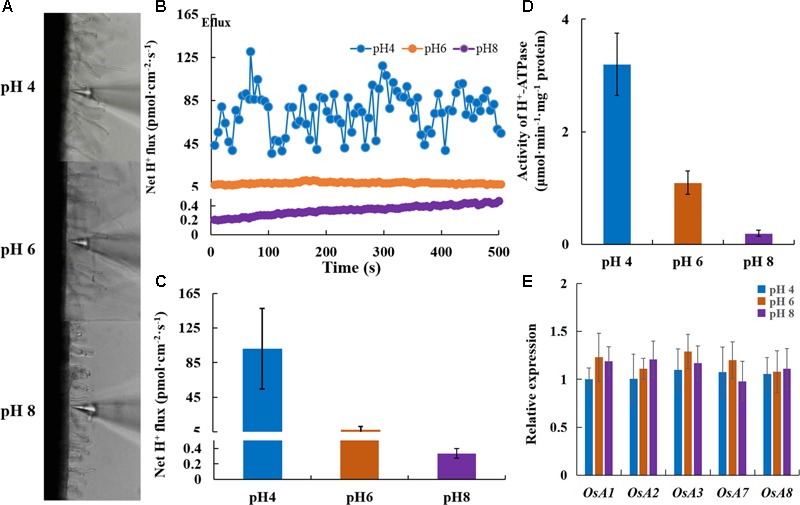
Net H^+^ flux rate of roots responding to different pH conditions. After 7 days of germination, Nipponbare (WT) plants were grown hydroponically for 3 days under different pH conditions. **(A)** The non-invasive micro-test technology (NMT) was used to measure the root H^+^ fluxes 10 mm from the root apex. **(B,C)** Net H^+^ flux rate of roots (WT) responding to different pH conditions (*n* = 8). **(D)** H^+^-ATPase activity in roots under different pH conditions. **(E)** Relative expression of the genes encoding H^+^-ATPase in roots. Values are the means and error bars denote the SE.

The elongation of the primary root requires cell wall acidification via PM H^+^-ATPase (Cosgrove, 2005; Sánchez-Rodríguez et al., 2010). Under normal pH, transgenic plants overexpressing *OsA8* (a rice PM H^+^-ATPase) showed a faster root elongation rate in 7-day-old seedlings and better growth than Nipponbare (WT) plants (**Figures 5A,D**). The root H^+^ efflux detected using the non-invasive micro-test technology revealed that overexpressing *OsA8* in plants enhanced the H^+^ excretion under pH 6 (**Figures 5B,C**), indicating positive effect of H^+^-ATPase on root elongation and plant growth. However, the OX-A8 plants displayed as severe growth inhibition as the WT plants, which showed short roots and a lower dry weight under alkaline stress (**Figures 5D–F**). We considered that the suppression of rice growth may be associated with PM H^+^-ATPase activity. Using the non-invasive micro-test technology (**Figure 6A**), we found that the H^+^ flux rate was extremely influenced by external pH (**Figure 6B**). The mean rate of H^+^ efflux in the root was decreased from 100 to 0.3 pmol⋅cm^-2^⋅s^-1^ as the pH was increased from 4 to 8 (**Figure 6C**). We measured the H^+^-ATPase activity in response to pH 4, 6, and 8, and the results showed that the H^+^-ATPase activity was greatly reduced as the external pH increased from 4 to 8 (**Figure 6D**). We investigated the expression level of plasma membrane H^+^-ATPase genes under alkaline stress and the results showed that the expression of *OsA1, OsA2, OsA3, OsA7* and *OsA8* genes was not significantly influenced by external pH (**Figure 6E**). These results suggest that the H^+^ efflux mediated by H^+^-ATPase was inhibited under alkaline stress, which may be post-transcriptionally regulated by the H^+^-ATPase activity level.

**FIGURE 7:**
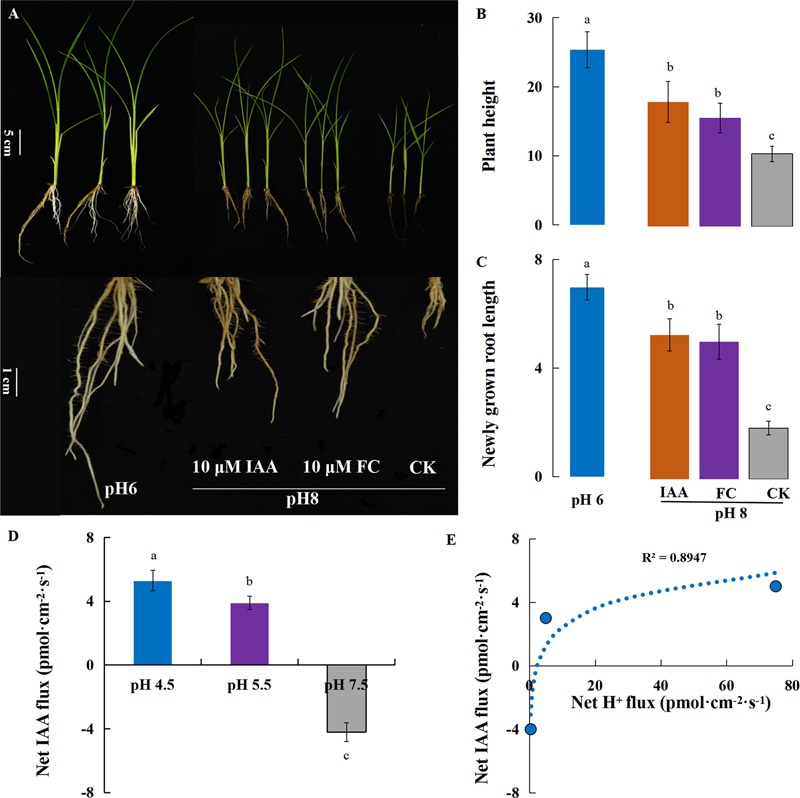
Rice plants under alkaline stress with auxin (IAA) and fusicoccin (FC) treatments. Plants (cv. Nipponbare) were subjected to alkaline stress (pH 8) with FC (a PM ATPase stimulator; 10 μM) or exogenous auxin (IAA; 10 μM) for 5 days in hydroponic culture. **(A)** The plant growth under alkaline stress (pH 8) with exogenous FC and IAA. **(B)** Plant height and **(C)** newly grown root length were investigated after 5 days of treatment. **(D)** Responses of the root net IAA fluxes rate to different external pH conditions. **(E)** The correlation between H^+^ flux rate and IAA flux rate. Values are the means (*n* = 6) and error bars denote the SE. Different letters represent statistical significance among treatments (*P* < 0.05).

Previous research reported that the interaction between PM H^+^-ATPase and 14-3-3 proteins was essential to the activation of H^+^-pump activity. Fusicoccin (FC), a phytotoxic metabolite produced by *Fusicoccum amygdali*, activates H^+^-ATPase activity by forming the H^+^-ATPase 14-3-3 complex that is stabilized by FC binding (Baunsgaard et al., 1998). Auxin promotes cell elongation by stimulating cell wall acidification that may be attributed to the auxin-induced activation of H^+^-ATPases according to the Acid Growth Theory. In the present study, rice growth was investigated under alkaline stress with exogenous auxin (IAA), the PM H^+^-ATPase inhibitor vanadate (NaVO_3_, VA) and the PM H^+^-ATPase enhancer fusicoccin (FC). Under the normal condition, the plant growth was inhibited by exogenous VA treatment and root elongation was almost completely inhibited (Supplementary Figure S4), indicating that H^+^-ATPases are required for root elongation. In comparison with the simple alkaline stress treatment, exogenous FC and IAA significantly improved primary root elongation and strengthened the tolerance of rice to alkaline stress (**Figures 7A–C**), indicating the H^+^-ATPase activity was regulated by protein modification under alkaline stress. The results showed that the IAA efflux was decreased at high pH, and the correlation analysis revealed a strongly positive correlation (*R*^2^= 0.89, *P* = 0.08) between the Net H^+^ and IAA flux rates on rice roots 10 mm from the root tip (**Figures 7D,E**).

### The Inhibition of H^+^-ATPase Activity Was Regulated by Ethylene

**FIGURE 8:**
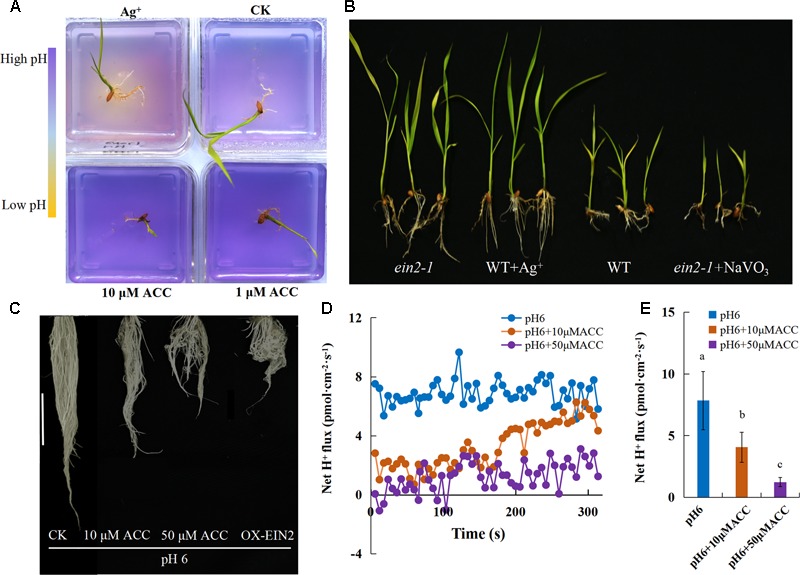
The involvement of ethylene in alkaline stress-induced inhibition of root H^+^-ATPase. **(A)** The initial pH of the medium was adjusted to 8 and the medium acidification was visualized using the pH indicator bromocresol purple (0.04 g L^-1^) for treatments at pH 8 with ACC (1 and 10 μM) and AgNO_3_ (10 μM). **(B)** WT (cv. Nipponbare) and mutant *ein2-1* plant growth under an alkaline condition (pH 8) with AgNO_3_ (10 μM) and NaVO_3_ (100 μM). **(C)** Root morphology and **(D,E)** Net H^+^ fluxes rate under a normal condition (pH 6) with ACC (10 and 50 μM). Values are the means (*n* = 8), and error bars denote the SE. Different letters represent statistical significance among treatments (*P* < 0.05).

These results demonstrated that the inhibition of rice growth was associated with ethylene production and H^+^-ATPase activity was reduced at high pH. We analyzed the relationship between ethylene signaling and H^+^-ATPase activity in alkaline stress-mediated root inhibition by pH indicator staining to visualize rhizosphere acidification. The initial pH of the media was adjusted to 8, appearing purple due to the pH indicator bromocresol purple (0.04 g L^-1^). When the medium turns acidic, it turns yellow due to the pH indicator. By applying an ethylene antagonist and ethylene precursor in the medium at pH 8, acidification was detected for the rhizosphere with exogenous 10 μM Ag^+^, an antagonist of ethylene perception, and almost no acidification was found in the rhizosphere with exogenous ACC (**Figure 8A**), suggesting that the acidification by H^+^-ATPase was negatively affected by ethylene. The rice growth was greatly recovered upon treatment with 10 μM Ag^+^ and the blocking the ethylene signal (*ein2-1*) under high pH stress (**Figure 8B**). The *ein2-1* mutants, which were less sensitive to alkaline stress than WT plants, displayed equally severe inhibition of growth as the WT plants under an alkaline condition with 100 μM NaVO_3_ (**Figure 8B** and Supplementary Figure S5). Under the normal pH, root growth and gravitropism were inhibited in the presence of exogenous ACC and the plants displayed curly roots (**Figure 8C**). We measured the root Net H^+^ flux rate and H^+^-ATPase activity with exogenous ACC, both of them were significantly decreased in the presence of ACC (**Figures 8D,E** and Supplementary Figure S6). These results verified that H^+^-ATPase is involved in ethylene-mediated inhibition of rice growth and ethylene acts as an upstream regulator that represses H^+^-ATPase activity.

## Discussion

High pH limits the survival of plants by disturbing numerous metabolic and physiological processes including ionic homeostasis, the ROS balance, and stability of the membrane system (Monshausen et al., 2007; Zhang and Mu, 2009). In this study, we observed severe inhibition of rice growth under alkaline stress, especially in the root elongation zone, including a shorter apical unbranched zone in the newly grown roots. In addition, the cell length in the unbranched zone was reduced in high pH and was further inhibited by exogenous ACC (**Figure 3**). Research suggested that excess aluminum and boron deficiency induce rapid production of ethylene, resulting in inhibition of root elongation (Sun et al., 2007; Martín-Rejano et al., 2011; González-Fontes et al., 2015). The biosynthesis of ethylene is regulated by 1-aminocyclopropane-1-carboxylic acid synthase (ACS) and 1-aminocyclopropane-1-carboxylic acid oxidase (ACO) (Argueso et al., 2007; Lyzenga et al., 2012). We found the genes encoding *ACS* and *ACO* were up-regulated by alkaline stress (**Figure 2**), and the plants displayed the same inhibition of root elongation upon 10 μM ACC treatment as under alkaline stress (**Figures 3A–F**). The rice plants growth was inhibited more by exogenous ACC under alkaline stress than under a normal condition, suggesting that ethylene biosynthesis was promoted by the up-regulation of *ACO* genes that catalyze ethylene formation with the ACC substrates. Furthermore, the *ein2-1* mutants were less sensitive to alkaline stress, but the *eto1-1* ethylene overproducing mutant was hypersensitive to alkaline stress (**Figures 4A–D**). Exogenous ACC inhibits cell elongation in the root elongation zone of *Arabidopsis* (Staal et al., 2011). We observed a significant inhibition in the cell elongation in rice roots under alkaline stress, and the inhibition was aggravated by the ethylene precursor ACC (**Figures 3C,F**). Our results provide direct evidence that ethylene is involved in alkaline stress-induced inhibition of rice root growth. However, Ag^+^ and the *ein2-1* mutants only partially alleviate root elongation inhibition under alkaline stress. So we conclude other factors other than ethylene are also involved in alkaline-mediated inhibition of rice.

PM H^+^-ATPase is necessary for the adaptation of plants to alkaline stress by proton-secretion (Palmgren, 2001; Liu and Guo, 2011). The low H^+^-ATPase activity was considered as a key factor that inhibited the root cell elongation under alkaline stress. Root elongation is inhibited within minutes by ACC, and the apoplast is alkalized concomitantly in the affected root (Staal et al., 2011), which suggests inactivation of H^+^-ATPase activity. We explored the relationship between ethylene and H^+^-ATPase activity by monitoring root medium acidification and the H^+^ efflux rate under exogenous Ag^+^ and ACC treatment. Ethylene negatively affected the root medium acidification (**Figure 8A**) and decreased the H^+^ efflux rate (**Figures 8D,E**). The root elongation of *ein2-1* mutants was less sensitive to alkaline stress and was restricted by NaVO_3_ (**Figure 8B** and Supplementary Figure S5). These results indicate that, acting as the upstream regulator, ethylene negatively affected H^+^-ATPase activity. The expression levels of plasma membrane H^+^-ATPase genes were not influenced (**Figure 6E**) and we found that the overexpression of *OsA8* in rice significantly promoted the root elongation at a normal pH (**Figure 5A**), and there was no effect on the adaption of rice to alkaline stress (**Figures 5D–F**), indicating that suppression of the proton efflux may occur through post-transcriptional regulation under alkaline stress. Activation of PM H^+^-ATPase by phosphorylation is important for the response to blue light (Kinoshita and Shimazaki, 1999) and aluminum stress (Shen et al., 2005). The tomato 14-3-3 Protein TFT4 was demonstrated to modulate H^+^ efflux in the response of root growth to alkaline stress (Xu et al., 2013). Fusicoccin (FC) can rapidly induce post-translational activation of the H^+^-ATPase by forming the H^+^-ATPase 14-3-3 complex, which is stabilized by FC binding (Oecking et al., 1997). We noted that fusicoccin (a PM ATPase enhancer) significantly improved the tolerance of rice to an alkaline condition (**Figures 7A–C**).

Auxin has also been suggested to have a role in regulating the activity of plasma membrane H^+^-ATPase, which determines the elongation of primary root via apoplastic pH regulation (Hager et al., 1991; Rober-Kleber et al., 2003; Barbez et al., 2017). In *Arabidopsis*, previous work found that ethylene modulates alkaline stress-mediated inhibition of root growth through stimulating expression of *AUX1*, as loss function of AUX1 led to root ethylene and alkalinity insensitivity (Rùzicka et al., 2007; Li et al., 2015). PIN2 (an auxin efflux transporter) activates plasma membrane H^+^-ATPase to release protons, which was necessary for adaptation of *Arabidopsis* to alkaline stress, and *pin2* mutants showed increased sensitivity to alkalinity (Xu et al., 2012). In our results, IAA efflux was decreased by high pH, and the root H^+^ efflux rate was closely associated with the IAA efflux rate (**Figures 7D,E**). Compared with the simple alkaline stress, primary root elongation and shoot growth were significantly improved upon treatment with IAA (**Figure 7C**). We suggest that ethylene may negatively regulate root H^+^-ATPase via transport-dependent redistribution of auxin under alkaline stress.

## Conclusion

Our results confirm that ethylene is involved in alkaline stress-mediated inhibition of plant growth, especially apical root elongation, providing evidence that control of the activity of plasma membrane H^+^-ATPase is a mechanism by which ethylene negatively regulates the H^+^-ATPase activity at the post-transcriptional level.

## Author Contributions

HaC conducted most of the experiments. QZ and HoC performed the transcriptional analysis. QZ and HoC participated in the proton flux assessment. FX designed and supervised the study. HaC analyzed the data and wrote the paper.

## Conflict of Interest Statement

The authors declare that the research was conducted in the absence of any commercial or financial relationships that could be construed as a potential conflict of interest.
