# Supplementary material for: Ethylene Mediates Alkaline-Induced Rice Growth Inhibition by Negatively Regulating Plasma Membrane H+-ATPase Activity in Roots
Source: Front Plant Sci. 2017 Oct 24;8:1839. doi: 10.3389/fpls.2017.01839 (PMC5660857; doi:10.3389/fpls.2017.01839)
Supplement: Supplementary file 1 [file Data_Sheet_1.DOCX]

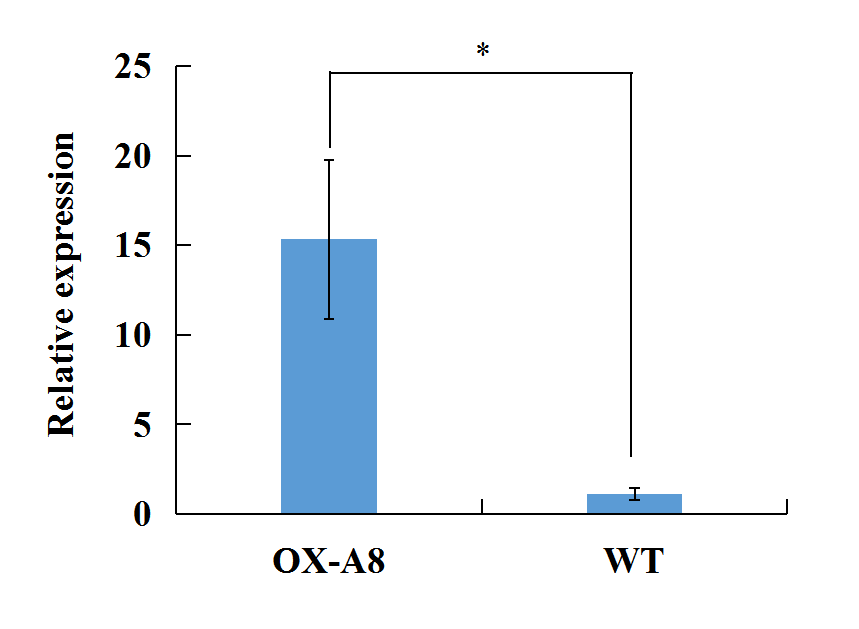


**Fig. S1** The expression of *OsA8* in [transgenic](C:/Users/apple/AppData/Local/youdao/dict/Application/7.2.0.0703/resultui/dict/?keyword=transgenic) [plant](C:/Users/apple/AppData/Local/youdao/dict/Application/7.2.0.0703/resultui/dict/?keyword=plant)s and WT plants*.*


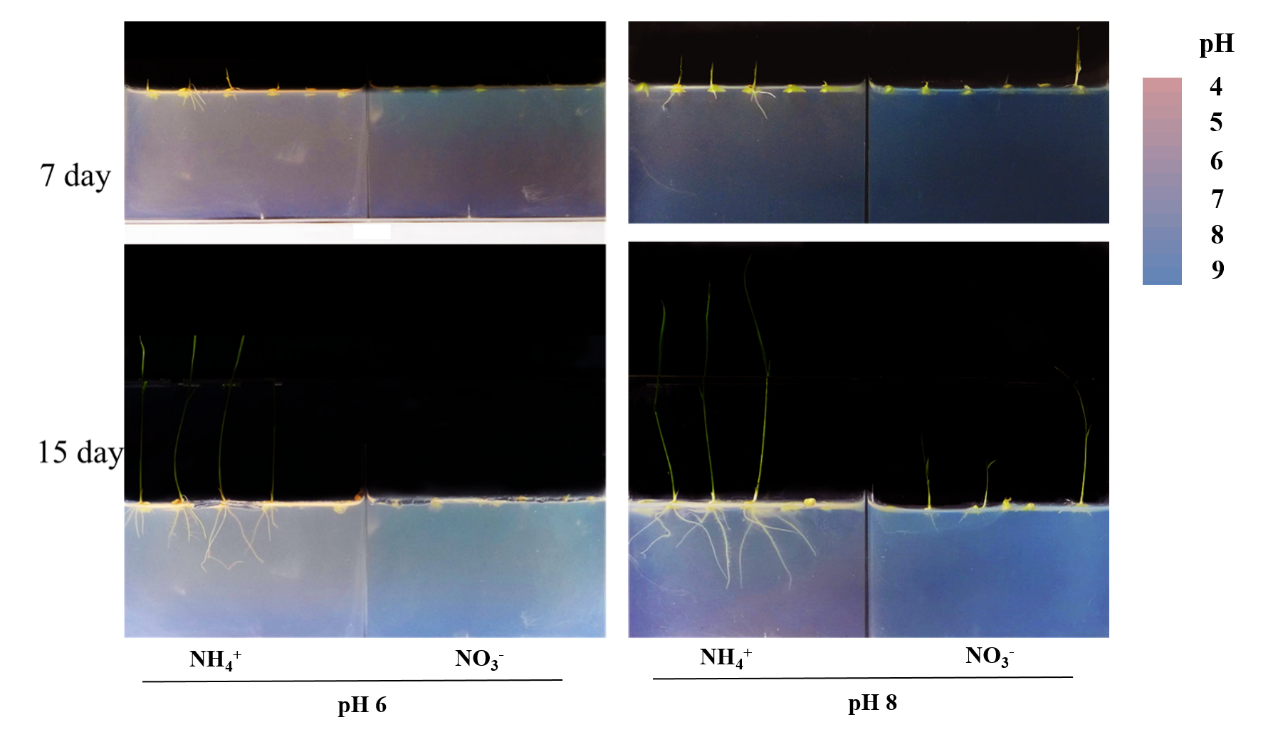


**Fig. S2** The response of rice (*cv.* Nipponbare) growth to NH_4_^+^ and NO_3_^-^ and pH changes of the agarose solid medium induced by NH_4_^+^ and NO_3_^-^. Medium supplemented with sole ammonium or nitrate nutrition was used to explore rice root growth and medium pH change with an initial pH of 6 or 8.


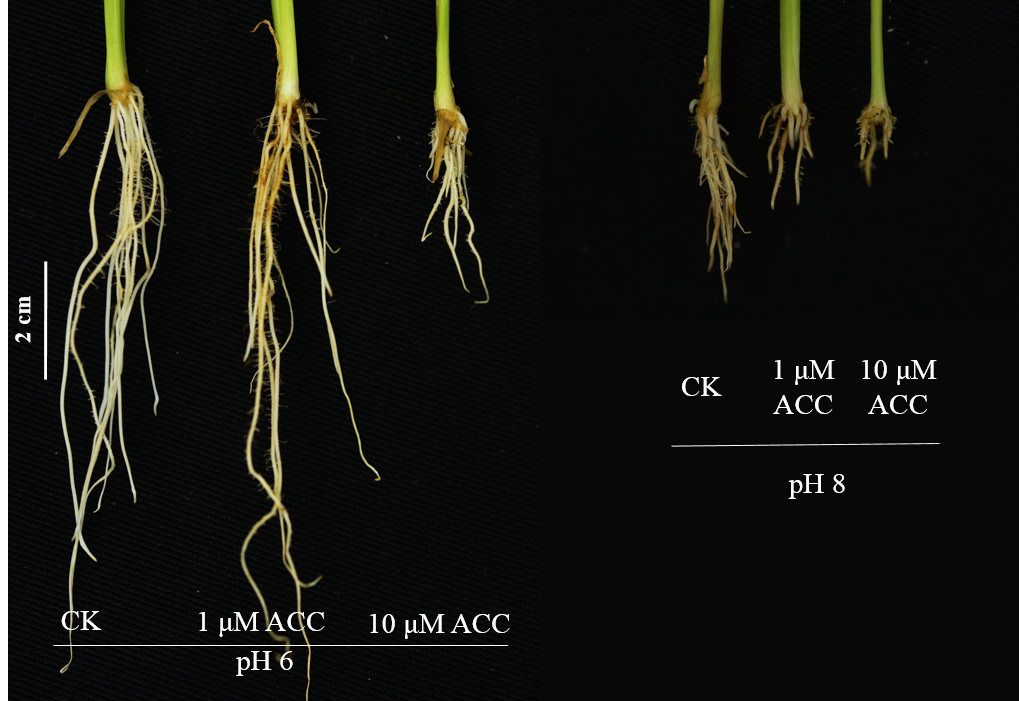


**Fig. S3** The newly grown root length under various pH conditions with different ACC treatments. After 7 days of germination, plants (*cv*. Nipponbare) were grown hydroponically for 7 days at varying pH and treated with 0, 1, or 10 μM ACC.


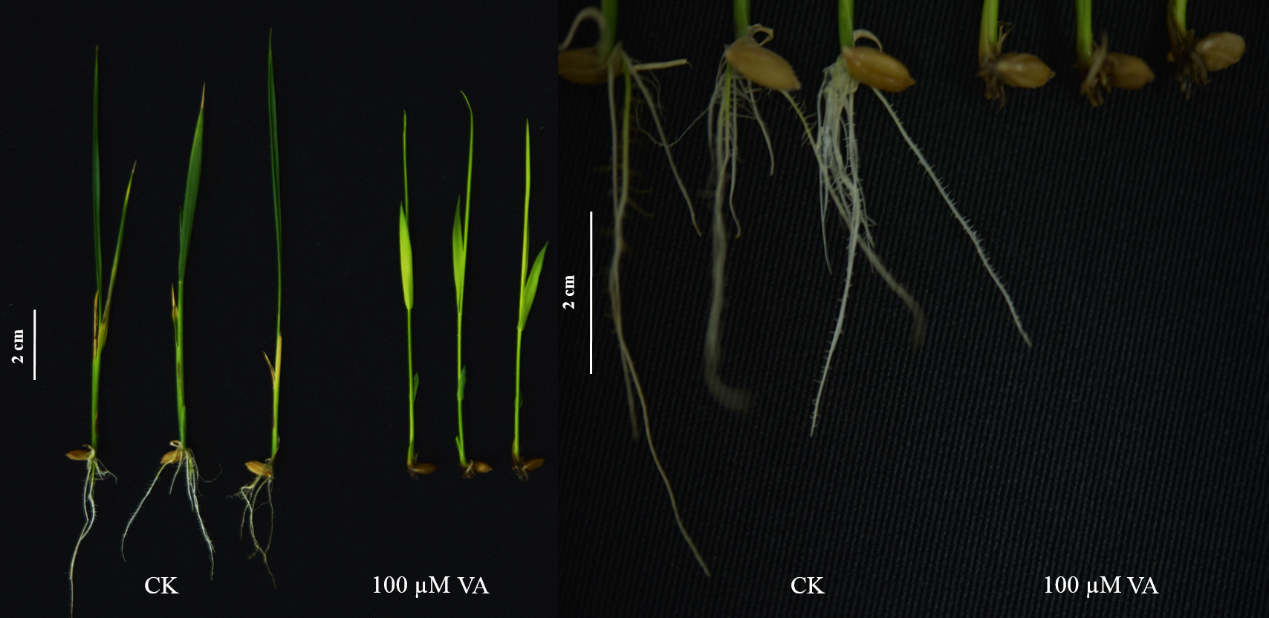


**Fig. S4** Plant growth and root elongation under a normal condition with the PM ATPase inhibitor vanadate (VA). The seeds (*cv*. Nipponbare) were surface sterilized for 15 min using 0.5% NaClO (w/v), rinsed completely with ultrapure water, and incubated at 30°C for 3 days before planting on medium containing one-half-strength MS at pH 6 for 7 days.


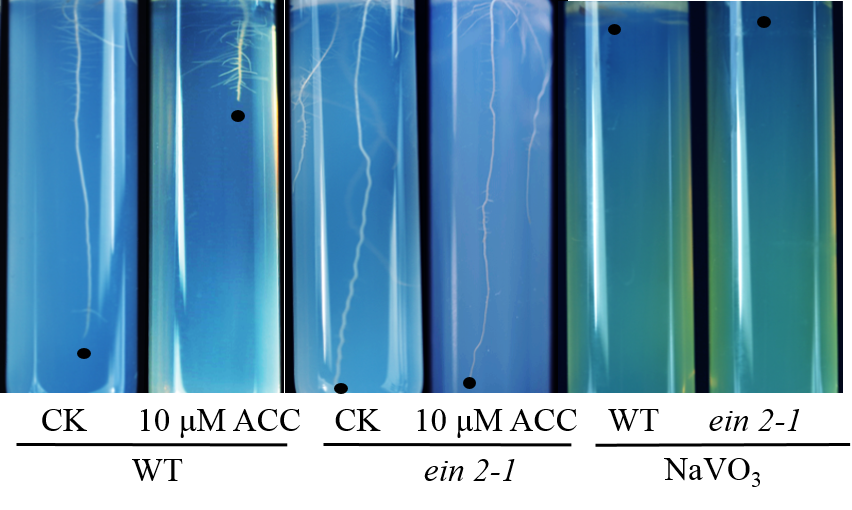


**Fig. S5** Root elongation of WT (*cv*. Nipponbare) and *ein2-1* plants under a normal condition with ACC and VA. The seeds were surface sterilized for 15 min using 0.5% NaClO (w/v), rinsed completely with ultrapure water, incubated at 30°C for 3 d, and planted on medium containing one-half-strength MS at pH 6 for 5 days.


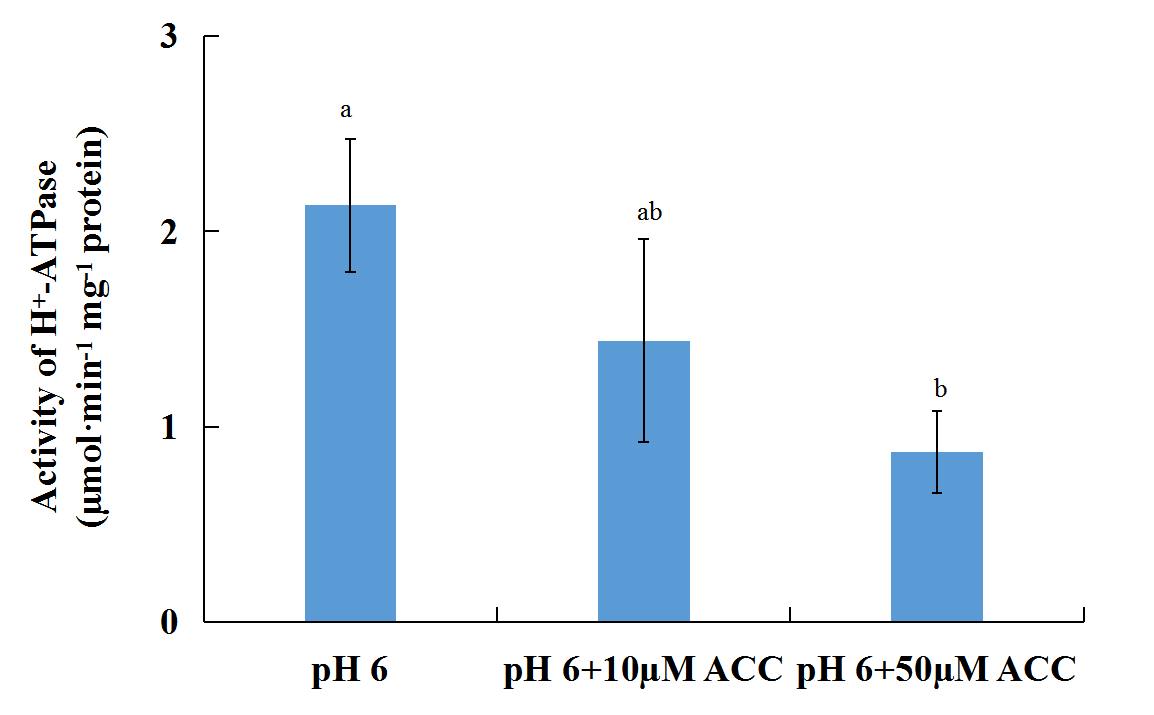


**Fig. S6** H^+^-ATPase activity in roots under a normal condition (pH 6) with ACC (10 µM, 50 µM). After 3 days of treatment in pH 6 solutions, the PM H^+^-ATPase activity was determined.

| **Supplemental Table S1.** PCR primer sequences | |
| --- | --- |
| **Gene** | **Primer** |
| OsACS1 | F-5'-CAGATTCCTTTCGTTTCTTGTAATCTT-3'  R-5'-AGACCATCGGGCATGCA-3' |
| OsACS2 | F-5'-CTGCTAATGCTGCGCCTTACT-3'  R-5'-CGACGATGCGATCGAGAAA-3' |
| OsACS3 | F-5'- GAGGCGAAGCTGAACATCTC-3'  R-5'- CATGTTGTTCTTGCTCCCATT-3' |
| OsACS4 | F-5'- AGCTGAGGCTGTGGGACA-3'  R-5'- GTGGCCAGGCTCATGTTC-3' |
| OsACS5 | F-5'- GCTTGGACACGCTGGATCTT-3'  R-5'- TTATTGCTGTTCTTGCTGCTG-3' |
| OsACS6 | F-5'- GGATGGTTCAGGTGTTGCTT-3'  R-5'- CCTGGCAAAGCAGTTATTCC-3' |
| OsACO1 | F-5'- GATAGCGTGTGTACCACAGCGACC-3'  R-5'- AGGTAGAAAACGCGAGCTGA-3' |
| OsACO2 | F-5'- AAGTCCATGGAAACCGAGAC-3'  R-5'- CCACAGTTCATGCACACACA-3' |
| OsACO3 | F-5'- GAGGTTCGTGTTCGAGGACT-3'  R-5'- CGCAGCCGTAGCTAGTGAAG-3' |
| OsACO4 | F-5'- GCATGGCCAACATTGCTC-3'  R-5'- GTTCGCCAGGGCTGCGAACC-3' |
| OsACO5 | F-5'- CGAGTACCCGGAGTACGTGTT-3'  R-5'- ATTTTGGCGCCTTGACGGCC-3' |
| OsACO7 | F-5'- GGACTACTACCAGGGCACCA-3'  R-5'- GATTAGCGCACGCGATTTTA-3' |
| OsA1 | F-5'- aatgttgcattgattttcagagg-3'  R-5'- caacgaagaaagcaaagacaagt -3' |
| OsA2 | F-5'- acttttgtttgtggtgatgaagg -3' |
|  | R-5'- atatgaccaaaccaatcaggaga -3' |
| OsA3 | F-5'- atgtgaagctaactttgggatga -3' |
|  | R-5'- caagttaaagcgaaacaggaaaa -3' |
| OsA7 | F-5'- tccagcttggatttttgctta-3' |
|  | R-5'- cctgcaaaaatattgcgtga -3' |
| OsA8 | F-5'- tgccattaaattgccaactg-3' |
|  | R-5'- agcgttcgatgaaaaactgaa-3' |
| Actin | F-5'- TGGCATCTCTCAGCACATTCC-3'  R-5'- TGCACAATGGATGGGTCAGA-3' |
